# Experiences, Beliefs, and Values of Patients with Chronic Pain Who Attended a Nurse-Led Program: A Descriptive Phenomenological Qualitative Study

**DOI:** 10.3390/nursrep15080269

**Published:** 2025-07-25

**Authors:** Jose Manuel Jimenez Martin, Angelines Morales Fernandez, Manuel Vergara Romero, Jose Miguel Morales Asencio

**Affiliations:** 1 Programa de Doctorado en Ciencias de la Salud, Universidad de Malaga, 29071 Malaga, Spain; jmanueljima@gmail.com; 2 Hospital Universitario Costa del Sol, 29603 Marbella, Spain; alaiamf@yahoo.es (A.M.F.); manuel.vergara.r.sspa@juntadeandalucia.es (M.V.R.); 3 Facultad de Ciencias de la Salud, Universidad de Malaga, 29071 Malaga, Spain; 4 Instituto de Investigación Biomedica de Malaga (IBIMA-Bionand) (Spain) de Ciencias de la Salud, Universidad de Malaga, 29071 Malaga, Spain

**Keywords:** chronic pain, personal narratives, quality of life, qualitative research

## Abstract

**Aim:** To explore the experiences, beliefs, and values of patients who participated in a two-arm randomized clinical trial assessing a nurse-led intervention program for chronic pain self-management, which demonstrated positive effects on pain reduction, depression, and anxiety, and on health-related quality of life 24 months after completion of the program. **Design:** Descriptive phenomenological qualitative study. **Methods:** Patients were recruited via telephone, informed about the study, and invited to participate in an individual interview at a place of their choice (hospital or home). All interviews were audiotaped, and an inductive thematic analysis was performed. **Results:** Seven interviews were carried out between both groups. Six emerging categories were found: effective relationship with the healthcare system, learning to live with pain, family and social support, behaviors regarding pain, resources for self-management, and concomitant determinants. **Conclusions:** Patients report key aspects that help us to understand the impact of this type of nurse-led group intervention: the intrinsic therapeutic effect of participating in the program itself, the ability to learn to live with pain, the importance of family and social support, the modification of pain-related behaviors, and the identification of resources for self-care. The findings highlight the need for gender-sensitive, individualized care approaches to chronic pain, addressing stigma and social context. Expanding community-based programs and supporting caregivers is essential, as is further research into gender roles, family dynamics, and work-related factors.

## 1. Introduction

The negative effects of chronic pain on the biological, psychological, and social dimensions of an individual are widely recognized [1]. The complexity of chronic pain lies in its multidimensional nature, independent of tissue damage [2], leading patients to experience feelings of invisibility, negativity, loneliness, or dominance of pain in their lives [3,4], which is why a multidimensional approach is essential for the successful care of these patients.

A metasynthesis [5] showed how people describe their experience of living with chronic pain based on five meta-themes: “the body as obstacle”, “invisible but real”, “disrupted sense of self”, “unpredictability”, and “keeping going”. Additionally, barriers to and facilitators of pain self-management have been explored in multiple studies, either from the perspectives of patients [6,7], nurses [8,9] or a combination of both [2]. In this regard, the interdisciplinary approach has proven to be the best option [10,11,12], placing nursing as a fundamental element in the development of self-care [13,14].

As a global strategy for non-pharmacological interventions, group therapies have emerged as the gold standard in pain self-management support [1,15,16], allowing patients to share their experiences, beliefs, and values related to their health and enabling them to identify both strengths and weaknesses in their coexistence with it. Far from referring only to pharmacological therapy [17], they give a special value to family, friends, diet, and lifestyle, etc. [16], or even postural health or distractions [6] as a means of reinforcement.

The impact of intervention groups has been evaluated in several experimental studies, with positive results for early return to work and reduction in medication [18,19], decreased suffering and increased self-understanding [15], acceptance of pain as an ongoing process [20], and feeling recognized as a person who is part of decision-making in health services [3].

However, there has been relatively little exploration of the perceptions and experiences of patients receiving these interventions, as the “post-intervention” follow-up period for these patients has not exceeded 12 months [13,18,19], limiting the ability to understand how patients integrate these interventions into their lives over time, or which elements may contribute most to sustained benefits. Moreover, there is limited research exploring how gender, family dynamics, or comorbidities intersect with the self-management process and influence long-term outcomes. Given that chronic pain often becomes a persistent and invisible companion, understanding how individuals live with and adapt to it over extended periods is crucial for designing interventions that are truly patient-centered. This lack of exploration hinders the identification of possible explanations for the long-term success of the interventions, as well as the identification of elements that could be enhanced, modified, improved, or eliminated to add value to them. The research question that guided this study was as follows: “What are the experiences, beliefs, and values that persist 24 months after participating in a nurse-led chronic pain self-management program?”

The general aim was to explore the experiences, beliefs, and values of patients who participated in a nurse-led intervention program for chronic pain self-management that showed positive effects on pain reduction, depression and anxiety, and on health-related quality of life [21] 24 months after completion of the program (the RCT was conducted from 2015 to the end of 2017). The intervention consisted of one individual session and six weekly 90 min group sessions led by an advanced practice nurse specializing in chronic pain. The program focused on enhancing self-esteem, fostering communication, increasing pain awareness, managing emotions and thoughts, promoting acceptance of health status, teaching Jacobson’s relaxation techniques, and encouraging health-seeking behaviors and decision-making [21].

The specific objectives were to understand post-intervention experiences of living with pain, the perceived value of the pain management program, the strategies acquired, and the reported changes in quality of life, to capture first-hand descriptions of patients’ experiences, while preserving their richness, and providing a comprehensive and deep insight into the patients’ lifeworld [22,23].

## 2. Materials and Methods

### 2.1. Design

A descriptive phenomenological qualitative study was used, following the COREQ reporting standard [24].

### 2.2. Theoretical Framework

Giorgi’s descriptive phenomenological approach [25] guided this study, to obtain in-depth accounts of participants’ lived experiences, drawing from Husserl’s philosophy that emphasizes grounding knowledge in the direct understanding of tangible phenomena, rather than in abstract theorizing.

### 2.3. Study Setting and Recruitment

The participants in this study were former participants in a nurse-led intervention program, which was evaluated in a randomized clinical trial described elsewhere [21]. After verification of the inclusion criteria, patients were recruited by telephone, informed about the study, and invited to participate in an individual interview at a place of their choice (hospital or home). Written informed consent was obtained, and patients consented to the use of a tape recorder during the session to facilitate the subsequent transcription process.

### 2.4. Inclusion and Exclusion Criteria

A purposeful sampling strategy was deployed, according to the following criteria: for the intervention group, patients were invited to participate in the groups that were attended in the last two thirds of the trial (patients in the first third were outside the 24-month post-intervention timeline for the data analysis in this study, the maximum time considered optimal for assessing the possible effect of the intervention), and patients corresponding to the same period in the control group. Individuals who showed limited discourse during the interview, despite consistent interviewer encouragement and facilitation, and those who chose not to participate voluntarily, were excluded. Sampling was conducted using the principle of data saturation.

### 2.5. Data Collection

A semi-structured interview with open-ended questions was prepared based on the meta-themes found by Crowe et al. [5] (Supplementary File S1). Only one patient chose to be interviewed in her own home, and the rest were interviewed in a designated office at the Hospital Universitario Costa del Sol. A specific room was prepared for this purpose, in order to create a calm and trusting environment that would encourage participants to express themselves openly, away from the potential influences of their family setting. In the particular case of the patient interviewed at home, she reported having an exceptional relationship with her family, feeling constantly supported and understood. Interviews were conducted by a male researcher, a registered nurse, with a master’s degree, who was not directly involved in patient care at the Pain Clinic. Data collection interviews took place between 2019 and 2021.

Considering this, it can be assumed that the interview setting did not significantly condition the content of participants’ narratives.

### 2.6. Data Analysis

Analysis was conducted following Giorgi’s five-step sequence [25]. All interviews and observations were transcribed verbatim and entered into qualitative data analysis software (MAXQDA 20.2). The first step involved the immersive reading of the entire dataset to gain a comprehensive understanding of the participants’ experiences. This was followed by a phenomenological reduction phase, in which the researchers applied bracketing to suspend personal assumptions, prior knowledge, and beliefs, including any personal experiences related to non-malignant chronic pain. Each member of the research team prepared a reflexive memo outlining “taken-for-granted” ideas about the phenomenon and the findings they might expect. These reflections were shared and discussed collectively at the outset of the analysis to promote reflexivity throughout data collection, analysis, and interpretation [26].

Next, the researchers identified and segmented relevant passages into meaning units, aiming to capture the essence of the lived experience of non-malignant chronic pain as described by the participants. These meaning units were synthesized into codes, categories, and overarching themes that represented the core of the phenomenon studied.

The coding process involved three iterative rounds. Initially, one researcher coded the data in MAXQDA using open coding. In subsequent rounds, the codes and categories were compared, refined, and grouped into broader themes through consensus meetings. A third round of joint analysis was conducted to ensure consistency across the dataset. All analytical decisions, including code development and category refinement, were documented in memos within MAXQDA to ensure traceability and transparency.

Finally, the identified themes were integrated into a coherent narrative describing the patients’ lived experiences with chronic non-malignant pain.

### 2.7. Ethical Considerations

The study was approved by the Research Ethics Committee of the Hospital Universitario Costa del Sol (MR2-CALIDO). Informed consent was obtained from all participants in the study.

### 2.8. Rigor and Reflexivity

The qualitative research quality criteria proposed by Guba and Lincoln were used to assess credibility, transferability, consistency, and confirmability as fundamental pillars [27]. The triangulation process of codes provided internal validity, not only for the assignment of codes to interviewees’ accounts, but also for obtaining feedback from participants and applying the saturation criterion. Anonymity and confidentiality guaranteed the authenticity of the discourses and were maintained before, during, and after the interviews and analysis. Interviewees were given verbal and written assurances that confidentiality would be preserved and that the information would be used for research purposes only. The presence of audio recording could have raised concern among interviewed subjects, so the reasons for its use were explained in detail. Credibility was also ensured by means of data collection with adequate time involvement, which allowed for exhaustive collection and sufficiently rich subsequent descriptions. Likewise, the analysis of disconfirming cases was carried out via comparison with subjects who had participated in the control group.

## 3. Results

A total of 25 patients were invited to participate (13 in the intervention group and 12 in the control group). Even though a meeting at their home was offered, the main reason why most of them rejected was the inconvenience of going to the hospital. Finally, seven interviews were carried out between both groups (five in the intervention group and two in the control group). The average duration of these interviews was 1 h 20 min.

The mean age of patients was 53 years. A total of three participants were male and four female. The median time that they had experienced chronic pain was over 84 months. The main characteristics of the participants are described in Table 1.

The patients describe their experiences, beliefs, and values regarding their life with pain, the significance of having access to the pain management program in the intervention group, the tools they acquired in it, and the changes they achieved to improve their quality of life. Their discourse, therefore, is organized around six main categories (composed of a total of 42 codes): effective relationship with the healthcare system, learning to live with pain, family and social support, behaviors regarding pain, resources for self-management, and concomitant determinants, each of which is related to a specific topic.

### 3.1. Effective Relationship with the Healthcare System

People with chronic pain use the healthcare system more frequently than the general population, which increases the usual consequences derived from their functioning. On the one hand, patients in the control group highlight waiting lists and inaccessibility as points to be improved.

- “…they told me that they would call me to put the device in and have it for 24 h, and I have been waiting 3 months for them to call me. So, it is not the doctor’s fault, but the long waiting list here…” (E7C).
- “…the problem is this, the waiting lists. For example, when you come here to the emergency room, you already fear it because in the emergency room you have to wait five hours to get a shot to get relief, and it’s not reasonable…” (E7C).
- “…and the only thing I would like is that there were more people who had access to this…” (E6C).

The intervention group, on the other hand, focuses more on the “lack of reinforcement”, a resource to turn to in case of acute need.

- “…or you may find yourself at a point where your family doctor does not have enough time to address your medication, or your pain…a phone number or something, a department where we can say at a certain moment, ‘Look, I have this problem, how do I deal with it?’ You know what I mean? And they can tell you, ‘Look, come to one of the sessions we have, listen a little bit of…’” (E2).
- “…it may be a relief for you to know that you can call for help at any time…” (E2).

In addition, the positive atmosphere of the group is highlighted as a new way of relating to the healthcare system—more enriching and resourceful—simply because of the receptivity and welcome it offers.

- “It wasn’t just a hospital visit, it felt more like a gathering, a chat between friends, which in turn gives a sense of comfort and builds greater trust in the pain unit.” (E2).
- “…but it was much nicer in terms of how you have to deal with pain, because there is a lot of distance between what the clinical consultation is and what the sessions were.” (E4).
- “…just knowing that you have to come on a certain day… and just thinking, ‘Right! Well, look, tomorrow I have to go to this’. Well, you got up in a different mood.” (E4).

In the same way, and as a result of the restructuring of thinking, this group manifests a new way of facing their daily lives, empowering themselves and taking control of their lives.

- “…I don’t want to wake up with pain as the first thing on my mind, there’s more to life than that. We’ve been taught that there’s more, there’s more out there…” (E2).
- “The answer is to change that mindset, to reverse it, what used to be meeting someone and say that I don’t feel well, I’m doing terrible and that I can’t…Well not anymore, now when I have a bad day I do the opposite, I take a shower, I get dressed, I comb my hair better, and I go out and I say that today no one will notice what is happening to me…” (E2).
- “…they taught us to not let the pain stop us from living because the pain will go away one day and we would have wasted that time…” (E1).

In general, they show their level of satisfaction with the program, highlighting the mutual support with people in the same health situation as a fundamental element for discovering intersubjective aspects that can become personal coping resources.

- “…because I learned that I am not the only one in pain, that there are many people in the same situation.” (E1).
- “…I mean, I think it’s (the improvement in pain) because of each other’s support and knowledge of each other’s different cases…” (E4).
- “I think the best thing is to share your experience so that others can listen to you and if you have achieved something, expose it so that others can also find the way…” (E2).

### 3.2. Learning to Live with Pain

The diversity of feelings that emerge in the interviews is notorious, which, moreover, are grouped differently according to the group to which they belong. On the one hand, patients in the intervention group identify more with suffering and the feeling of being misunderstood as suffering from something “invisible”.

- “…if you learn to live with pain, you learn to live with people’s suffering.” (E1).
- “…people thought that by being there and by learning to live in a different way, others would say that they don’t have as much pain…”; “…because it’s something invisible.” (E2).

On the other hand, the control group uses terms related to frustration and resignation due to not being able to develop a full and independent life.

- “…because you are always in a bad mood, taking it out on others who are not to blame, because that is what pain brings and not being able to develop normal activities during the day, leads you to be under stress and tension to the point that is not normal…” (E7C).
- “…because it can be really hard to constantly listen to someone complaining, since there is nothing you can do about it, and that’s the only thing that affects the person who is listening to you, especially if they care about you…” (E6C).

An interesting finding to highlight at this point has to do with the aptitudes of the patients. Initially, both groups converge on a perceived lack of irreversible self-efficacy.

- “…the neck, I am already convinced that I will never be able to move my neck properly…” (E6C).
- “…because it has affected me to the point where I can’t do anything…I was a person who was very active at work, now I don’t even see myself capable of making the bed…” (E7C).
- “…you get stuck in a loop of saying I can’t, I can’t, I can’t…it’s always I can’t.” (E2).
- “…because before you came, I had already looked at things by myself, because I started at 35, so I said, ‘I have a long way to go, because life today is long, and it is not very pleasant to be in constant pain…’”. (E2).

However, the program seems to have helped participants to “discover” a new way of coping with those situations that had previously produced such a lack of self-efficacy in the intervention group.

- “…before all I could think about was taking the pills and being with the heat blanket back and forth, well now I say, ‘No, I have to go out…’” (E1).
- “…you have to learn to live, you have to get up and say well, if you stay in bed, the same as I told you before, if you stay at home, you will have pain and if you go to work you will have pain…you have to be very positive.” (E1).
- “…that the pain can’t get to you, you can handle it, it’s the realization that the power of the mind is everything.” (E2).

In addition, the patients in this group emphasize how accepting that the pain will not go away and that they will have to live with it is the step prior to overcoming and developing new coping mechanisms for their health situation that will allow them to develop as people. Patients verbalize an “inner dialog” with their own pain in their daily life.

- “…while I, for example, assume what I have and my limitations and so on.” (E4).
- “…that’s the most important thing, to learn that it’s true that you’re going to carry the pain wherever you go”; “…the pain is going to be there, but you have to learn to live with it…and it’s like it becomes a part of you. You say, ‘Well, it’s true, it’s going to be there with me all the time, what nonsense…because anyway, whether you’re working, whether you’re at home, however you are, the pain, the day it has to be there, it’s there.’” (E1).
- “…I wanted to live, I wanted to enjoy myself…”; “And, besides, I am able to go out, before I could not say let’s go out to a party…” (E2).
- “…today I am in pain, but look, since I am having lunch with my children today, let’s not dwell on that right now…” (E5).

Related to the above (coping), women show more interest in learning to manage their processes and adapt to their chronic situation. In addition, when it comes to finding coping mechanisms for their situation, the mother’s role plays an important part.

- “…we will learn something from this, for sure; she will learn something from having her illness and we will also learn, and that is the part I take…”; “I want everything I do in life to have a purpose…and if I mess up, I learn from the fact that I mess up, of course I’m human…and I make mistakes, I learn from the them, correct them and try to improve…” (E1).
- “I am also a mother, my son has entered university, he needs me financially so, a mother does everything she can and much more for a child, then I put up with much more.”; “…I had a son, then I looked for my own way, then I looked for many therapies.” (E2).
- “…today I am in pain, but look, since today I am having lunch with my children, let’s not dwell on that right now…” (E5).

Men, however, focus more on overcoming physical activities and functional capacity.

- “…so I make a great effort to do my daily exercise routine, I ride my bike, I use 1 kg weights for my exercises. I’m not a person who, despite the discomfort, let’s say, will not confront it head-on.” (E7C).
- “I was a marathon runner, and when I got sick, I could no longer run marathons, I realize that, and well, I miss it, but that does not prevent me from continuing to live.” (E4).

### 3.3. Family and Social Support

Family support, understood as the family’s understanding of the situation of their loved one, feeling useful in different situations (work, with themselves, etc.), and being able to share different scenarios with friends or with the family itself (socialization), is a strategy highlighted by the participants as very positive for strengthening the social sphere.

- “…it’s also because my family is very supportive, my wife and my daughter, they always look after me.” (E4).
- “…and after 5 years, going back to work, feeling useful, earning money…well, all that has an impact, of course.” (E3).
- “…doing something, that makes you feel productive and helps you mitigate the pain.” (E4).
- “…I take it very well, personally I take it very well. My family is doing well, they have learned to live with my things…” (E2).

Conversely, the health of these family members also plays a limiting role, taking time away from the patient’s own self-care.

- “I had some serious problems at home, I had to give up swimming, I had to give up… forget a little bit about myself…”; “…I’ve been suffering a lot for more than two years… my mother was diagnosed with a pretty big breast cancer, she had to undergo breast surgery… then my father had prostate cancer…”; “…also a lot of family depression, family problems.” (E1).

### 3.4. Behaviors Regarding Pain

Based on each person’s personal experience with their pain, there are different levels of knowledge about how to deal with it, sometimes even creating negative anticipatory thoughts. This leads to common behaviors in both groups, such as concealment (pretending not to be in pain).

- “…but it wasn’t until I understood, or someone said it to me, that I realized that the more stress, the more pain. Then the key was to break the toxic loop.” (E6C).
- “…you think it’s going to hurt and you get very nervous…” (E4).
- “…I keep the pain to myself, and I don’t tell anyone about it and I go on with my pace of life…”; “it’s not because I don’t tell anyone, you can’t let a comment slip out, can you? But no, for me it is normal.” (E6C).

On the other hand, the acceptance that they will go through better and worse stages in terms of pain intensity (cycles of pain experience) is more related to the intervention group, thus them forming a more proactive coping attitude compared to the control group.

- “…I’ve had cyclical pain…”; “…then, when it subsides for a while, I’m fine, with less pain…then I’ve had cycles where the pain has increased…” (E4).
- “…then I wake up and I start thinking about the pain and I say, ‘No, stop thinking about it’, and I start to relax.” (E1).
- “…the other pain depends on the weather, the weather in summer is a little better, but in winter the weather is terrible…” (E7C).

### 3.5. Resources for Self-Management

Similarly, there is another behavior called “resources for self-management”, which emphasizes the active role of patients in finding and using the available resources. In the intervention group, and mainly in women, physical activity and the correct use of medication stand out as the main strategies.

- “…because I was swimming, I was doing little things to improve my back, right?” (E1).
- “I have done tai chi, I have gone to the pool, I have done yoga, I do Pilates” (E2).
- “…then I learned very well how to take medication, which I didn’t manage very well at all, the steps of medication, how to manage them, how to combine them, that helped me.” (E2).
- “…and so far, I only take Tramadol when I need it, because there are days when the humidity and other things cause me pain…in my back, my right hip, and then is when I take it…but I don’t abuse it like I used to…” (E3).

The control group, on the other hand, shows a more passive attitude towards the problem at hand.

- “…I mean, there are times when you have to ask for help, if you can’t move, you can’t move…” (E6C).
- “…when I take my medication, then I have breakfast, and half an hour later I can move a little better, using a cane…” (E7C).

Both groups seem to agree that distracting themselves with other types of activities is beneficial for pain control.

- “…and when that happens, I distract my mind until I get sleepy again, and when I get sleepy it seems like the pain also goes away.” (E1).
- “…being distracted helps mitigate the pain, meeting people or going on a trip for example, in the sense that you’re not always dealing with your own thing…I don’t get bored, I’m always thinking of something to do or practicing things that I’m doing, but with the feeling of being distracted and mitigating the pain, which we also learned here.” (E4).
- “…while you are eating, while you are with your loved ones, or while you are doing something, it (the pain) goes away…with your grandchildren, it disappears… Well, it’s those pain-free moments that will make you move on.” (E5).
- “…things and projects that force me to focus on other things, not on twiddling my thumbs, because if I keep twiddling my thumbs, the pain will come for sure.” (E6C).
- “…when you relax, you don’t think about the pain and the pain goes down, and that’s the strategy I have, to stop and rest a little bit, and then I say, ‘Well, let’s go on and don’t think too much about the pain.’” (E7C).

### 3.6. Concomitant Determinants

This refers to the various situations that, although they are not an intrinsic part of the pain itself, in one way or another influence the people who suffer from it. The “comorbidities” of the patients themselves thus constitute one of the main modulators of their health condition (pain), negatively and directly influencing its control. We find, among others, visual limitations due to ophthalmological problems; the need to lead a quiet and effortless life due to a recurrent aneurysm; difficulties in ambulation and overweight that affect the autonomy of the person; problems at the emotional level (such as anxiety); and even other chronic diseases such as diabetes.

- “…but just like this retinal detachment that limits you…” (E4).
- “I had an aneurysm, they started to examine me and I had another one, so my health is… (describes the characteristics a little) … so of course I have to have a life where I can’t make efforts, and then that’s it.” (E4).
- “Afterwards, things start appearing over time, as in my feet, and now they have sent me some kind of a small insole because I couldn’t walk…” (E1).
- “…I already had a hip problem, I was about to undergo surgery…”; “…I remember that I even came with crutches and everything because I couldn’t even walk…and well, apart from the back problem…” (E3).
- “…but in the weight, because you can see that my legs also bear more weight, when I move around at work, I also notice it, I am not as agile as I used to be, the weight makes me not have that agility…” (E1).
- “But I am a very anxious person, very…I have suffered from anxiety.” (E1).
- “…what happens is that there are medications that they cannot prescribe for me because, for example, when I’ve had infiltrations, my blood sugar goes up a lot, so I have to control my blood sugar, but the internist said that it was better to control my blood sugar and just relieve the pain a little bit, so there I am, struggling between pain and diabetes…” (E7C).

Finally, an overly demanding work environment is also a negative influence, although this finding was not reported by the control group.

- “Well, when I had the surgery and started having so many problems, working was unfeasible with what I had at that time, I had to stop working…” (E2).
- “…I’d been out of work for 5 years, my back problems plus the hip…” (E3).
- “…of course that limits you a lot, especially for work and normal life…” (E4).

## 4. Discussion

The aim of this study was to explore the experiences, beliefs, and values of patients with chronic pain, in subjects with diverse quality of life and mental health situations, according to their experience in the participation of a nurse-led group intervention for non-malignant chronic pain.

The clinical trial showed significant effects on the experimental group participants regarding their perceived pain, as well as in perceived physical and mental health. A particularly significant finding from this qualitative assay is the ability of the group intervention to foster an environment for sharing experiences with other patients facing similar challenges, ultimately aiding in the development of new coping mechanisms. This finding has been similarly identified in previous studies [28], and a systematic review describes the “supportive environment” as an external factor that influences the development of coping strategies for these patients [29] and facilitates adherence to this type of intervention [30]. Moreover, in some studies [31], patients rated participation in the program as a non-negotiable step, a fact that may be related to the feeling of being part of the decision-making process regarding their health, described in this study and categorized as “person-centered care”.

Stenberg et al. [32], from a review of qualitative studies, identify some motivational elements for chronic pain self-care when interactions with peers occur, including the improvement of competence (through information exchange, mutual support, and re-evaluation through comparison with peers), autonomy (promoting decision-making and the consideration of options, recognition of the participants’ perspective, emphasis on self-responsibility), and “connection and belonging” (relatedness) to the group. Possibly, these elements explain the participants’ very clear perception of the impact of the group intervention and confirm the importance of this intervention modality with this type of patient.

Another major contribution of the program has to do with the behavioral resources for self-management, understood as all those activities/behaviors that patients use in their daily lives to control their pain, serving as a catalyst for positive life changes. This aligns with the findings of the study by Ruiz-Romero [33], which highlighted improved pain and sleep management, a more positive outlook on life, and enhanced coping as the primary personal benefits experienced by the participants.

Considering that chronic pain becomes a cross-cutting issue in patients’ lives, it is not surprising that some mention that the role of medication in the whole process appears in their discourses. In a recent publication [34], participants describe how opioid treatment helps them to take control of their lives again and that, despite suffering stigmatization for it, they are able to accept that it is part of their new health condition.

Similarly, patients in this study also reflect on medication, although they focus more on the information received in the program about how to take it, possible side effects, and rescue doses, etc. However, they also emphasize the role of the family as a facilitating element in pain management, an unconditional support that reflects the family’s understanding of the situation their loved one is going through.

Our study showed important differences in self-efficacy between patients who participated in the program and those who did not. The change in perceived self-efficacy reported by patients in the first group is a finding that may explain why such differential results in perceived pain and perceived mental and physical health were obtained in the clinical trial. In quantitative studies, self-efficacy has been identified as a predictor of physical function in people with chronic pain, although there is little evidence regarding disability, mental health, or quality of life [35]. In contrast, in systematic reviews of qualitative studies, self-efficacy has revealed an improved sense of ability to overcome difficulties and to change belief patterns regarding pain, as well as being modifiable via educational interventions, provided that hey focus on relevant goals for patients [29].

Regarding the factors that negatively influence pain self-management, the literature shows some variety. For some, functional limitations, pain, and “flare ups” [29]. For others, the “perception that pain is created by the person” or the “belief that not everyone will understand the meaning of the interventions”, etc. [30]. All of these are far removed from the situations described by the patients in this study as barriers to pain self-management, referred to as “concomitant determinants”. This concept includes both the patient’s own comorbidities, which are not related to pain but have a negative impact on pain management (diabetes, retinal detachment, etc.), and those of their relatives, which force them to put self-care on the back burner in order to assume the role of caregiver (especially those requiring daily care, such as cancer). Similarly, a work environment that is too demanding or not adapted to their health condition also acts as a barrier to self-care.

Accessibility to the healthcare system is an element described as an obstacle by the patients in this study, which is consistent with the dissatisfaction with waiting times defined in Torralba’s work [36]. However, a potential solution to this problem may lie in self-care programs tailored to chronic pain management. According to the systematic review by Spink [29], these programs have been shown to promote compliance and participation by increasing individuals’ confidence and sense of security, largely due to their easy accessibility.

Learning to live with pain encompasses a range of feelings and behaviors for patients. It is interesting to note that one of them (this “inner dialog with pain”) coincides with the results of a much earlier study [37], which suggests that this behavior is possibly quite common among patients with chronic pain.

Our study found differences in adaptive behaviors in favor of women, particularly regarding appropriate medication use and engagement in physical activity as a strategy to improve quality of life. Several studies have shown that gender influences both the experience of pain and coping behaviors, with women reporting higher prevalence and employing a wider range of adaptive strategies, as also observed in our findings. While some biological factors may partially explain sex-based differences in chronic pain, more consistent associations have been found between psychological factors—such as anxiety, depression, and perceived quality of life—and chronic pain in women. However, sociocultural aspects of gender, including gender identity and roles, remain underexplored, and may significantly shape how individuals cope with pain. Moreover, gendered beliefs and expectations can introduce biases in how professionals assess and manage pain in women [38].

On the other hand, the difficulty of leading a full and independent life, described in this study in the control group, which could resemble the term described in the literature as “A life ruled by pain” [13], seems to be a point to improve in order to achieve results such as “Active involvement to achieve a meaningful life” [15], “Striving for a meaningful life” [39], or “Live a life, not only survive” [13].

Another major barrier to improved self-management of chronic pain has to do with the complexity of assessing it. The literature outlines, consistently with this study, how patients describe it as something “invisible”, a condition that is not seen and therefore hinders its credibility, leading to behaviors such as concealment [1,4,5].

In this regard, a recent study [40] indicated that improving communication in the family environment leads to a climate of understanding of the health situation of these patients. This fact is also in line with the findings of this study, which suggest how the family becomes a fundamental element of support, thus constituting a facilitator in the management of chronic pain.

### Study Limitations

Firstly, the number of possible interviews in the control group could have been influenced by the lack of a previous relationship with the interviewer (the intervention group had built such a relationship during the six sessions of the program, so the interviewer was not a “stranger” to them when they were invited to participate in the qualitative phase), as well as by the restrictions that marked the COVID-19 pandemic (the patients from the experimental group had been interviewed in a close previous period). The period during which the study was conducted, which coincided with the COVID-19 pandemic, generated significant fear among patients with chronic conditions regarding mobility and seeking healthcare. This had a considerable impact on recruitment and represents a limitation of the results.

Unlike participants in the intervention group, control patients did not maintain close contact with healthcare professionals during the study, which may have contributed to their greater reluctance to engage in interviews. This hesitation could also be partially explained by a poorer self-perception of their health status, potentially fostering a lack of interest in participating at this stage of the research. In the intervention group, however, the main reasons for declining participation appeared to be logistical, mainly related to transportation difficulties, such as the lack of suitable public transport connections or the absence of a family member to accompany them to the hospital. Furthermore, given the personal and social context of these patients—individuals who frequently live with chronic pain, often concealing it due to feelings of misunderstanding by those in their immediate environment—the decision to decline an in-home interview is also understandable. Alternative interview modalities, such as telephone or video calls, were intentionally avoided for the following reasons: It was considered unlikely that these formats would provide a sufficiently calm and trustworthy environment to encourage patients to share detailed and meaningful narratives. Non-verbal communication, which was systematically captured in the interview transcripts, would have been lost. Most patients reported difficulties in using such technological tools, which could have further compromised participation and data quality.

On the other hand, although the impact of family support on the outcomes has been mentioned, there is a noticeable absence of extensive discussion by patients when compared to the previous literature.

As for the lack of reports from the control group on the possible influence of the work environment on their situation, this could be the result of the small sample and low saturation of the data.

## 5. Conclusions

This qualitative study gathers the experiences of patients with non-malignant chronic pain who participated in a nurse-led intervention program that was shown to be effective in a previous experimental study. In their discourse, key aspects emerge that help us to understand some of the elements provided by this type of nurse-led group intervention: the intrinsic therapeutic effect of participating in the program itself, the ability to learn to live with pain, the importance of family and social support, the modification of pain-related behaviors, and the identification of resources for self-care.

This study reinforces the value of nurse-led group interventions as a strategy to enhance chronic pain self-management. These programs promote peer support, foster emotional validation, and facilitate the adoption of adaptive behaviors such as appropriate medication use and physical activity. Incorporating structured self-management education into routine care is essential to improve patients’ autonomy and quality of life.

The findings also underscore the need for gender-sensitive approaches, recognizing that coping strategies and perceived barriers may differ between men and women. Clinicians should address the invisibility and stigma surrounding chronic pain and ensure that care is tailored to individual needs and social contexts.

At the policy level, expanding community-based programs can help reduce access barriers and reinforce continuity of care. Supporting informal caregivers and integrating flexible care pathways are also key to improving outcomes. Finally, further research is needed to explore how gender roles, family dynamics, and work-related factors influence chronic pain experiences and management.

## Figures and Tables

**Table 1:** Individual patient profiles in the experimental and control groups.

**Experimental Group**							
ID	Age	Gender	Pain Etiology	Duration of Pain (Months)	Months Treated in Pain Unit	Occupational Status	Educational Level
E1	50	Female	Mixed	36	0	Employed	Primary education
E2	43	Female	Neuropathic	96	84	Unemployed	University degree
E3	43	Male	Neuropathic	156	36	Disabled	Primary education
E4	51	Male	Neuropathic	120	0	Sick leave	Primary education
E5	65	Female	Musculoskeletal	480	48	Retired	University degree
**Control Group**							
	Age	Gender	Pain Etiology	Duration of Pain (Months)	Months Treated in Pain Unit	Occupational Status	Educational Level
E6C	66	Female	Musculoskeletal	10	2	Retired	Primary education
E7C	57	Male	Musculoskeletal	180	2	Employed	Primary education

## Data Availability

The data presented in this study are available on request from the corresponding author due to privacy and ethical restrictions.

## Supplementary Materials

The following supporting information can be downloaded at: https://www.mdpi.com/article/10.3390/nursrep15080269/s1, File S1: Interview guide.

## Public Involvement Statement

No public involvement in any aspect of this research.

## Guidelines and Standards Statement

This manuscript was drafted in accordance with the Consolidated Criteria for Reporting Qualitative Research (COREQ), available at https://www.equator-network.org/reporting-guidelines/coreq/, (accessed on 23 May 2025).

## References

[1] Furnes B., Natvig G.K., Dysvik E. (2015). Suffering and transition strategies in adult patients attending a chronic pain management programme. J. Clin. Nurs..

[2] Gjesdal K., Dysvik E., Furnes B. (2019). Mind the Gaps: A Qualitative Study Combining Patients’ and Nurses’ Reflections on Pain Care. SAGE Open Nurs..

[3] Gjesdal K., Dysvik E., Furnes B. (2018). Living with chronic pain: Patients’ experiences with healthcare services in Norway. Nurs. Open.

[4] Ojala T., Häkkinen A., Karppinen J., Sipilä K., Suutama T., Piirainen A. (2015). Chronic pain affects the whole person--a phenomenological study. Disabil. Rehabil..

[5] Crowe M., Whitehead L., Seaton P., Jordan J., Mccall C., Maskill V., Trip H. (2017). Qualitative meta-synthesis: The experience of chronic pain across conditions. J. Adv. Nurs..

[6] Alvarado-García A.M., Salazar-Maya Á.M. (2017). Everything is valid in chronic pain: Interventions by older adults for pain relief. Enferm. Clin..

[7] Devan H., Hale L., Hempel D., Saipe B., Perry M.A. (2018). What Works and Does Not Work in a Self-Management Intervention for People With Chronic Pain? Qualitative Systematic Review and Meta-Synthesis. Phys. Ther..

[8] Bergeron D.A., Bourgault P., Gallagher F. (2015). Knowledge and Beliefs about Chronic Non Cancer Pain Management for Family Medicine Group Nurses. Pain Manag. Nurs..

[9] Ladak S.S.J., McPhee C., Muscat M., Robinson S., Kastanias P., Snaith K., Elkhouri M., Shobbrook C. (2013). The journey of the pain resource nurse in improving pain management practices: Understanding role implementation. Pain Manag. Nurs..

[10] Andersen L.N., Stochkendahl M.J., Roessler K.K. (2022). En route to flourishing—A longitudinal mixed methods study of long-term unemployed citizens in an interdisciplinary rehabilitation program. BMC Public Health.

[11] Falkhamn L.M., Stenberg G., Enthoven P., Stålnacke B.M. (2023). Interdisciplinary Multimodal Pain Rehabilitation in Patients with Chronic Musculoskeletal Pain in Primary Care—A Cohort Study from the Swedish Quality Registry for Pain Rehabilitation (SQRP). Int. J. Environ. Res. Public Health.

[12] Stewart C., Schofield P., Elliott A.M., Torrance N., Leveille S. (2014). What do we mean by «older adults’ persistent pain self-management»? A concept analysis. Pain Med..

[13] Hållstam A., Stålnacke B.M., Svensen C., Löfgren M. (2015). «Change is possible»: Patients’ experience of a multimodal chronic pain rehabilitation programme. J. Rehabil. Med..

[14] Lukewich J., Mann E., VanDenKerkhof E., Tranmer J. (2015). Self-management support for chronic pain in primary care: A cross-sectional study of patient experiences and nursing roles. J. Adv. Nurs..

[15] Dysvik E., Kvaløy J.T., Furnes B. (2014). A mixed-method study exploring suffering and alleviation in participants attending a chronic pain management programme. J. Clin. Nurs..

[16] Takai Y., Yamamoto-Mitani N., Abe Y., Suzuki M. (2015). Literature review of pain management for people with chronic pain. Jpn. J. Nurs. Sci..

[17] Berlemont C. (2017). The non-pharmacological management of chronic pain. Soins Rev. Ref. Infirm..

[18] Chu M.C., Law R.K.Y., Cheung L.C.T., Ma M.L., Tse E.Y.W., Wong T.C.M., Chen P.P. (2015). Pain management programme for Chinese patients: A 10-year outcome review. Hong Kong Med. J..

[19] Ehrenborg C., Gustafsson S., Archenholtz B. (2014). Long-term effect in ADL after an interdisciplinary rehabilitation programme for WAD patients: A mixed-method study for deeper understanding of participants’ programme experiences. Disabil. Rehabil..

[20] Pietilä-Holmner E., Stålnacke B.M., Enthoven P., Stenberg G. (2018). «The acceptance» of living with chronic pain—An ongoing process: A qualitative study of patient experiences of multimodal rehabilitation in primary care. J. Rehabil. Med..

[21] Morales-Fernández Á., Jimenez-Martín J.M., Morales-Asencio J.M., Vergara-Romero M., Mora-Bandera A.M., Aranda-Gallardo M., Canca-Sanchez J.C. (2021). Impact of a nurse-led intervention on quality of life in patients with chronic non-malignant pain: An open randomized controlled trial. J. Adv. Nurs..

[22] Holloway I. (2016). Qualitative Research in Nursing and Healthcare.

[23] Matua G.A., Van Der Wal D.M. (2015). Differentiating Between Descriptive and Interpretive Phenomenological Research Approaches. Nurse Researcher (2014+). https://www.proquest.com/docview/1784854054/abstract/85F8710286394902PQ/1.

[24] Tong A., Sainsbury P., Craig J. (2007). Consolidated criteria for reporting qualitative research (COREQ): A 32-item checklist for interviews and focus groups. Int. J. Qual. Health Care.

[25] Giorgi A. (2009). The Descriptive Phenomenological Method in Psychology; A Modified Husserlian Approach.

[26] Hamill C., Sinclair H. (2010). Bracketing—Practical considerations in Husserlian phenomenological research. Nurse Res..

[27] Guba E.G., Lincoln Y.S. (1989). Fourth Generation Evaluation.

[28] Robinson V., King R., Ryan C.G., Martin D.J. (2016). A qualitative exploration of people’s experiences of pain neurophysiological education for chronic pain: The importance of relevance for the individual. Man. Ther..

[29] Spink A., Wagner I., Orrock P. (2021). Common reported barriers and facilitators for self-management in adults with chronic musculoskeletal pain: A systematic review of qualitative studies. Musculoskelet. Sci. Pract..

[30] Rizzo R.R.N., Wand B.M., Leake H.B., O’Hagan E.T., Bagg M.K., Bunzli S., Traeger A.C., Gustin S.M., Moseley G.L., Sharma S. (2023). «My Back is Fit for Movement»: A Qualitative Study Alongside a Randomized Controlled Trial for Chronic Low Back Pain. J. Pain.

[31] Finlay K.A., Elander J. (2016). Reflecting the transition from pain management services to chronic pain support group attendance: An interpretative phenomenological analysis. Br. J. Health Psychol..

[32] Stenberg N., Gillison F., Rodham K. (2022). How do peer support interventions for the self-management of chronic pain, support basic psychological needs? A systematic review and framework synthesis using self-determination theory. Patient Educ. Couns..

[33] Ruiz-Romero M.V., Guerra-Martín M.D., Álvarez-Tellado L., Sánchez-Villar E., Arroyo-Rodríguez A., Sánchez-Gutiérrez M.C. (2021). Non-drug treatments for chronic non-malignant pain: Patients’ perceptions. Anales del Sistema Sanitario de Navarra.

[34] Ljungvall H., Rhodin A., Wagner S., Zetterberg H., Åsenlöf P. (2020). «My life is under control with these medications»: An interpretative phenomenological analysis of managing chronic pain with opioids. BMC Musculoskelet. Disord..

[35] Hayward R., Stynes S. (2021). Self-efficacy as a prognostic factor and treatment moderator in chronic musculoskeletal pain patients attending pain management programmes: A systematic review: Musculoskeletal Care. Musculoskelet. Care.

[36] Torralba A., Miquel A., Darba J. (2014). Situación actual del dolor crónico en España: Iniciativa «Pain Proposal». Rev. Soc. Esp. Dolor.

[37] Thomas S.P. (2000). A phenomenologic study of chronic pain. West. J. Nurs. Res..

[38] Keogh E. (2025). Sex, gender, and pain: Evidence and knowledge gaps. Curr. Opin. Psychol..

[39] Dysvik E., Furnes B. (2018). Living a meaningful life with chronic pain—Further follow-up. Clin. Case Rep..

[40] Rønne P.F., Esbensen B.A., Brødsgaard A., Biering-Sørensen B., Hansen C.A. (2023). Patients’ and Family Members’ Experiences of a Novel Nurse-Led Intervention Using Family Conversations Targeting Families Afflicted by Chronic Non-Cancer Pain. J. Pain Res..

