# Supplementary material for: Experiences, Beliefs, and Values of Patients with Chronic Pain Who Attended a Nurse-Led Program: A Descriptive Phenomenological Qualitative Study
Source: Nurs Rep. 2025 Jul 25;15(8):269. doi: 10.3390/nursrep15080269 (PMC12389680; doi:10.3390/nursrep15080269)
Supplement: Supplementary file 1 [file nursrep-15-00269-s001.zip › nursrep-3712497-supplementary.pdf]

Interview guide

1. Physical Health

Intervention group: How did you feel during the program and afterward regarding your physical health? If you noticed an improvement, what do you think contributed to this change?

Control group: How have you felt regarding your physical health over the past few months?

Both groups: How do you currently feel about your body?

2. Mental Health

Intervention group: How did you feel during the program and afterward regarding your mental health and overall wellbeing? If you noticed an improvement, what do you think contributed to this change?

Control group: How have you felt regarding your mental health and wellbeing over the past few months?

Both groups: Has your way of thinking or feeling about pain changed? If so, how? What has changed in your life in this regard?

3. Depression/Anxiety

Both groups: How have you felt in relation to depression or anxiety? Why do you think this happened? What do you believe made this possible?

4. Pain Evolution

Intervention group: After evaluating the program, we found positive results regarding the core issue: chronic pain. How would you explain this difference? What do you think this improvement is due to?

Control group: How has your experience of pain been over the past few months?

Both groups: What strategies have you used to keep going despite the challenges of living with pain? How has your relationship with others been, considering that your condition is not always visible to them?

5. Program Evaluation

Intervention group: Could you describe, in general terms, how the program has influenced your quality of life over this time? How useful do you think attending the program has been for you? Would you change or add anything to the sessions?

Were there any expectations you had about the program that were not met?

6. Final Reflections

Both groups: Is there anything else you would like to add? Any questions or reflections?
